# Applicability of the adjusted graded prognostic assessment for lung cancer with brain metastases using molecular markers (Lung‐molGPA) in a Chinese cohort: A retrospective study of multiple institutions

**DOI:** 10.1002/cam4.3485

**Published:** 2020-10-07

**Authors:** Tingyou Zhang, Yu Zhang, Lin Zhou, Shanshan Deng, Meijuan Huang, Yuncong Liu, Yongmei Liu, Youlin Gong, Jiang Zhu, Jianxin Xue, Yuju Bai, Hu Ma, Yan Zhang, Min Yu, Yanying Li, Yongsheng Wang, Bingwen Zou, Xiaojuan Zhou, Weigang Xiu, Feifei Na, Yong Xu, Feng Peng, Jin Wang, You Lu

**Affiliations:** ^1^ Department of Thoracic Oncology Cancer Centre Sichuan University West China Hospital Chengdu Sichuan P.R. China; ^2^ Department of Thoracic Oncology Zunyi Medical University NO.2 Affiliated Hospital Zunyi, Guizhou P.R. China; ^3^ Department of Oncology Guizhou Provincial People’s Hospital Guiyang, Guizhou P.R. China

**Keywords:** brain metastases, gene mutation, GPA, lung cancer, prognostic factors

## Abstract

**Background:**

In this era of precision medicine, prognostic heterogeneity is an important feature of patients with non‐small cell lung cancer (NSCLC) with brain metastases (BM). This multi‐institutional study is aimed to verify the applicability of the adjusted Lung‐molGPA model for NSCLC with BM in a Chinese cohort.

**Methods:**

This retrospective study included 1903 patients at three hospitals in Southwest China. The performance of the Lung‐molGPA model was compared with that of the adjusted DS‐GPA model in terms of estimating the survival of NSCLC with BM.

**Results:**

The median OS of this patient cohort was 27.0 months, and the adenocarcinoma survived longer than the non‐adenocarcinoma (28.0 months vs 18.7 months, *p* < 0.001). The adjusted Lung‐molGPA model was more accurate in predicting survival of adenocarcinoma patients than the adjusted DS‐GPA model (C‐index: 0.615 vs 0.571), and it was not suitable for predicting survival of non‐adenocarcinoma patients (*p* = 0.286, 1.5‐2.0 vs 2.5‐3.0; *p* = 0.410, 2.5‐3.0 vs 3.5‐4.0).

**Conclusions:**

The adjusted Lung‐molGPA model is better than the DS‐GPA model in predicting the prognosis of adenocarcinoma patients. However, it failed to estimate the prognosis for non‐adenocarcinoma patients.

## Graphical abstract

In the era of precision medicine, as rising healthcare costs of cancers, it has become essential for the society as a whole to predict the prognosis of NSCLC with BM, whose survival outcomes present heterogeneity even though new treatments strategies such as SRS, molecular target and immune therapy are being developed. Based on the Lung‐molGPA model, we generated an adjusted Lung‐molGPA model. It is aimed to verify the applicability of the adjusted Lung‐molGPA model for NSCLC with BM in a Chinese cohort that a retrospective study included 1903 patients at three hospitals in Southwest China. To our knowledge, this is the first multi‐center study about the applicability of adjusted Lung‐molGPA and DS‐GPA models in a Chinese cohort that the adjusted Lung‐molGPA model is better than the DS‐GPA model in predicting the prognosis of adenocarcinoma.

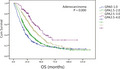

## 1 INTRODUCTION

Lung cancer is the major type of cancer and the major cause of cancer‐related mortality in China and in the rest of the world; non‐small cell lung cancer (NSCLC) accounts for 85% of these cases.^1^, ^2^ Lung cancer commonly metastasizes to the brain, patients with brain metastases (BM) only have median survival 1‐2 months in the natural course. In the era of precision medicine, more treatments are available for NSCLC with BM, due to the rapid developments in molecular target therapy and advances in radiation technology.^3^ It is interesting to note that there has been no significant survival benefit in patients with NSCLC and BM undergoing stereotactic radiosurgery (SRS) combined with anti‐PD‐1 therapies.^4^ Considering in the current climate of rising health‐care costs of cancers, it has become essential for the society as a whole to predict the prognosis of NSCLC with BM, whose survival outcomes present heterogeneity even though new treatments strategies such as SRS, molecular target, and immune therapy are being developed.

The most widely used tools for estimating survival in BM during the last decades include basic score for BM (BS‐BM), recursive partitioning analysis (RPA), and the graded prognostic assessment index (GPA).^5^, ^6^, ^7^ However, these tools do not just analyze lung cancer, and do not provide specific prognoses. Moreover, even after using these models, there is still a chance of BM recurrence after treatment in 60% to 70% of the patients.^8^ Sperduto et al. have developed a diagnosis‐specific prognostic factor index (DS‐GPA) that takes into consideration age, Karnofsky performance status (KPS), extracranial metastases (ECM), and BM numbers.^9^ Rades et al. have established a series of prognostic evaluation systems for BM of lung cancer with radiation.^10^, ^11^, ^12^ However, these models ignored the effect of driver gene mutation, a known prognostic factor, on the survival time of NSCLC. Therefore, Chen et al. developed an adjusted prognosis analysis (APA) model for evaluating individuals initially diagnosed with NSCLC and BM, and included the following six prognostic factors: KPS, age, smoking history (replaced by epidermal growth factor receptor (EGFR) mutation in APA 2), local treatment of intracranial metastases, EGFR‐tyrosine kinase inhibitor (TKI) treatment, and chemotherapy,^8^ which seems to be more advantageous than RPA and GPA. Most importantly, Sperduto et al. renewed the graded prognostic assessment for NSCLC with BM using molecular Markers (Lung‐molGPA) on the basis of DS‐GPA. Lung‐molGPA is a user‐friendly tool that may facilitate clinical decision making and appropriate stratification of future clinical trials.^13^ Nieder et al confirmed the validity of the lung‐molGPA in a retrospective study that included a German and Norwegian cohort treated with individualized care, but the median survival was shorter in 6 of 7 prognostic strata than the study of Sperduto.^14^ Li et al. proved the applicability of the Lung‐molGPA for accurately predicting the overall survival (OS) in a Northern Chinese cohort using the clinical data set of lung adenocarcinoma patients with BM. However, they identified that the independent prognostic factors were not entirely consist with the study of Sperduto.^15^ In a similar study on 1184 Eastern Chinese patients with NSCLC and BM, Fan et al. found that Lung‐molGPA can precisely estimate the survival outcomes of the subgroup of gene variation, although it did not perform well in wild type.^16^ Nevertheless, these studies were single‐center retrospective studies on Chinese and European populations, and their conclusions were slightly distinct from those observed using Sperduto's lung‐molGPA model. Therefore, more studies are needed to predict the prognosis so as to help doctors to make better therapeutic decisions and clinical trial stratification, and to promote rational allocation of medical resources.

Though the incidence and mortality of lung cancer is second only to Eastern China, there are no studies estimating the prognosis of NSCLC with BM in Southwest China.^17^ To our knowledge, this is the first multicenter study about the applicability of adjusted Lung‐molGPA and DS‐GPA models in a Southwestern Chinese cohort. This study retrospectively collected clinical and follow‐up data of 1903 NSCLC and BM cases. Additionally, the correlation between body mass index (BMI) and survival of Chinese population with NSCLC coupled to BM was analyzed first.

## 2 MATERIALS AND METHODS

### 2.1 Data set

This study was approved by the ethics committee of participating medical institution, Sichuan University West China Hospital, Zunyi Medical University Affiliated Hospital, and Guizhou Provincial People's Hospital. Considering the medical records were obtained from previous clinical diagnosis and treatment, and exemption from informed consent would not adversely affect the patients’ rights and health, the requirement for informed consent waived upon approval of the ethics committee.

We created a multi‐institutional retrospective database, including 1903 patients with primary NSCLC and newly diagnosed BM between 1 January 2008 and 31 May 2018 at Sichuan University West China Hospital, Zunyi Medical University Affiliated Hospital, and Guizhou Provincial People's Hospital. Those patients were excluded, including: (1) with multiple malignant tumors, small cell lung cancer (SCLC), neuroendocrine lung cancer, lung sarcoma, and mixed lung cancer; (2) with meningeal metastasis (MM) and BM along with MM were excluded; (3) only received best supportive care. Histological subtype was ascertained according the lung tumor classification criteria of the World Health Organization (WHO). This present study only included in patients with complete clinicopathological data, containing patient age, sex, smoking status, KPS, BMI, T and N stage, ECM, ECM organ numbers, BM numbers, histological subtype, and gene mutation status.

Till last follow‐up on 1 August 2019, 452 patients were still surviving, and 143 ones could not be traced; follow‐up loss rate was 7.5%. Among the 1903 cases, the follow‐up information regarding 317 patients was obtained using medical records or telephone calls, and for 1586 patients, information was collected using the resident identity information system of Huichuan District Public Security Sub‐bureau of Zunyi city, Guizhou province.

### 2.2 Analyses of prognostic factors and stratification

This study evaluated the correlation between prognostic factors and OS using univariate and multivariate analyses, and stratified the cases by referring to the criteria of the adjusted DS‐GPA and Lung‐molGPA models (See Table 1 for detail). Prognostic factors were analyzed, including patient age, sex, smoking status, KPS, BMI, T and N stage, ECM, ECM organ numbers, BM numbers, NSCLC subtype, and gene alteration status. Type of treatment was not considered because the goal of a prognostic model was to assess survival prior to treatment. However, patients received chemotherapy, TKI, and immunotherapy were 64.1%, 42.7%, 1.1%, respectively. In particular, patients received local brain therapy was 55.6%, 30.8% whole‐brain radiotherapy (WBRT), 14.3% stereotactic radiosurgery (SRS), 4.9% surgery, 1.9% WBRT + SRS, 1.8% WBRT + surgery, 1.7% SRS + surgery, and two patients received WBRT + SRS + surgery.

**Table 1:** Baseline characteristics included in adjusted Lung‐molGPA and DS‐GPA for NSCLC with BM at diagnosis

Prognostic factor	GPA Scoring Criteria			DS‐GPA^a^	Lung‐molGPA^a^
	0	0.5	1		
Age, y	≥57	<57	NA	_____	_____
KPS	<70	70‐80	≥90	_____	_____
ECM	Present	NA	Absent	_____	_____
BM, No.	>4	1‐4	NA	_____	_____
Gene status^a^	EGFR neg/unk and ALK neg/unk	NA	EGFR pos or ALK pos	NA	_____
Total	NA	NA	NA	_____	_____

Abbreviations: BM, brain metastases; DS‐diagnosis specific; ECM, extracranial metastases; GPA, graded prognostic assessment; KPS, Karnofsky Performance Status; Lung‐mol, NSCLC with BM using molecular Markers; NA, not applicable; neg/unk, negative or unknown; NO, number; NSCLC, non‐small cell lung cancer; pos, positive.

^a^ Evaluating clinician completes this column.

### 2.3 Statistical analysis

OS, as the primary end point, measured from the time of first diagnosis of BM‐by imaging‐till death by any cause or till end of last the follow‐up. Correlation between prognostic factors and survival analyzed by log‐rank (Mantel‐Cox) test for univariate analyses and Cox regression model for the multivariate analyses. Hazard ratios (HRs) and their 95% confidence intervals (CIs) were reported. Stratified analysis was performed using the log‐rank (Mantel‐Cox) test. The model performance was assessed by Harrell's concordance index (C‐index). *p* value <0.05 was considered statistically significant. All analyses were performed using the SPSS 22.0 software (SAS Institute, Cary, NC, USA) and the R software (version 3.3.1).

## 3 RESULTS

### 3.1 Demographics and clinical characteristics

There were 1903 cases with age between 20 and 84 years, and a median age of 57 years. In this cohort, 965 patients (50.7%) were above 57 years, 1126 individuals were men (59.2%), 924 individuals (48.6%) had a smoking history, and 228 individuals (12.0%) had not even quit smoking till the time of BM diagnosis. Of the total 1903 patients, 1085 individuals (57.0%) had KPS ≥90, and only 179 cases (9.4%) were less than 70. This study included 127 patients (6.7%) with malnutrition and 538 individuals (28.3%) with overweight. The vast majority cases were adenocarcinoma (N = 1588, 83.4%), and most patients were T and N stage in advanced. There were 1393 patients (73.2%) with ECM, and 986 cases (51.8%) with 1‐2 ECM organ numbers. There were 1182 individuals (62.1%) exhibited 1‐4 brain lesions and 815 patients (42.8%) presented with EGFR mutation or anaplastic lymphoma kinase (ALK) fusion. (See Table 2 for detail).

**Table 2:** Patients characteristics

Parameter	NO.	Percent (%)
Gender		
Male	1126	59.2
Female	777	40.8
Age (y)		
<57	938	49.3
≥57	965	50.7
Smoking		
Never	979	51.4
Ever	696	36.6
Current	228	12.0
BMI (kg/m^2^)		
<18.5	127	6.7
18.5‐23.9	1131	59.4
≥24.0	538	28.3
Missing	107	5.6
KPS		
≥90	1085	57.0
70‐80	639	33.6
<70	179	9.4
Histology type		
Adeno	1588	83.4
Non‐adeno	315	16.6
T‐staging		
T_0_ or T_X_	62	3.3
T_1‐_T_2_	591	31.1
T_3‐_T_4_	1250	65.7
N‐staging		
N_0_ or N_X_	361	19.0
N_1_	127	6.6
N_2_‐N_3_	1415	74.4
ECM		
Absent	510	26.8
Present	1393	73.2
ECM organ, NO.		
1‐2	986	51.8
≥3	407	21.4
Missing	510	26.8
BM, No.		
1‐4	1182	62.1
>4	721	37.9
Gene status		
EGFR/ALK neg	460	24.2
EGFR/ALK unk	628	33.0
EGFR or ALK pos	815	42.8

Abbreviations: Adeno, adenocarcinoma; BM, brain metastases; BMI, body mass index; ECM, extracranial metastases; KPS, Karnofsky Performance Status; neg, negative; NO, number; Non‐adeno, non‐adenocarcinoma; pos, positive.

Patients with NSCLC and BM had a median survival of 27.0 months from the time of initial diagnosis and the adenocarcinoma had a longer median OS than the non‐adenocarcinoma (28.0 months vs 18.7 months, *p* < 0.001). Univariate analysis revealed that female, KPS ≥90, BMI ≥24.0, adenocarcinoma, EGFR or ALK positive, significantly decreased the death risk of patients, but ECM (especially organ numbers ≥3), BM numbers ≥4, N2~N3, and smoking (especially current smoking) increased it. Multivariate analysis revealed that smoking, BMI, KPS, ECM organ numbers, BM numbers, histological type, N‐staging, and gene alteration were independent prognostic factors for NSCLC with BM. Interestingly, age and T‐staging were correlated with survival in neither univariate nor multivariate analysis in the current study (See Table 3 for detail).

**Table 3:** Univariate and multivariate analysis of prognostic factors for overall survival

Parameter	Median OS(m)	Univariate analysis		Multivariate analysis	
		HR (95% CI)	*p*‐value	HR (95% CI)	*p*‐value
Gender					
Male	24.4	1.00			
Female	29.8	0.77 (0.69‐0.87)	<0.001		
Age (y)					
≥57	25.6	1.00			
<57	28.3	0.92 (0.83‐1.03)	0.133		
Smoking					
Never	29.5	1.00		1.00	
Ever	24.4	1.21 (1.08‐1.36)	<0.001	1.12 (0.94‐1.33)	0.210
Current	18.3	1.67 (1.41‐1.98)	<0.001	1.41 (1.14‐1.75)	0.002
BMI(kg/m^2^)					
<18.5	20.8	1.00		1.00	
18.5‐23.9	26.5	0.86 (0.70‐1.07)	0.175	0.88 (0.71‐1.10)	0.259
≥24.0	29.3	0.74 (0.59, 0.93)	0.009	0.77 (0.61‐0.96)	0.022
KPS					
≥90	30.7	1.00		1.00	
70‐80	21.7	1.46 (1.30‐1.64)	<0.001	1.28 (1.13‐1.45)	<0.001
<70	18.8	1.47 (1.22‐1.76)	<0.001	1.32 (1.08‐1.61)	0.006
Histology type					
Adeno	28.0	1.00		1.00	
Non‐adeno	18.7	1.54 (1.33‐1.78)	<0.001	1.42 (1.21‐1.66)	<0.001
T‐staging					
T_0_ or T_X_	29.6	1.00			
T_1‐_T_2_	31.5	0.80 (0.58‐1.10)	0.164		
T_3‐_T_4_	24.5	1.11 (0.81‐1.51)	0.516		
N‐staging					
N_0_ or N_X_	35.1	1.00		1.00	
N_1_	33.8	0.84 (0.65‐1.09)	0.191	0.85 (0.65‐1.11)	0.233
N_2‐_N_3_	24.4	1.32 (1.14‐1.52)	<0.001	1.18 (1.01‐1.37)	0.033
ECM					
Absent	35.1	1.00			
Present	24.4	1.59 (1.40‐1.81)	<0.001		
ECM organ,NO.					
0	35.1	1.00		1.00	
1‐2	26.9	1.44 (1.26‐1.65)	<0.001	1.49 (1.28‐1.72)	<0.001
≥3	18.8	1.99 (1.69‐2.33)	<0.001	1.96 (1.63‐2.35)	<0.001
BM,No.					
1‐4	29.6	1.00		1.00	
>4	22.6	1.38 (1.23‐1.54)	<0.001	1.15 (1.02‐1.30)	0.019
Gene status					
EGFR/ALK neg	20.5	1.00		1.00	
EGFR/ALK unk	21.4	1.04 (0.90‐1.19)	0.602	0.96 (0.83‐1.12)	0.629
EGFR or ALK pos	33.1	0.64 (0.56‐0.74)	<0.001	0.65 (0.56, 0.76)	<0.001

Abbreviations: Adeno, adenocarcinoma; BM, brain metastases; BMI, body mass index; ECM, extracranial metastases; KPS, Karnofsky Performance Status; m, months; MS:median survival; neg, negative; NO, number; Non‐adeno, non‐adenocarcinoma; pos, positive.

### 3.2 Survival based on the adjusted DS‐GPA and Lung‐molGPA model stratification for NSCLC with BM in different histological types

Contrasting to the DS‐GPA and Lung‐molGPA models developed by Sperduto,^13^ that used 70 years as the cutoff, the age cutoff value was adjusted as 57 years (median age) in this study, because only 183 (9.6%) patients were over 70 years (Detailed description is shown in Table 1).

**Figure 1:**
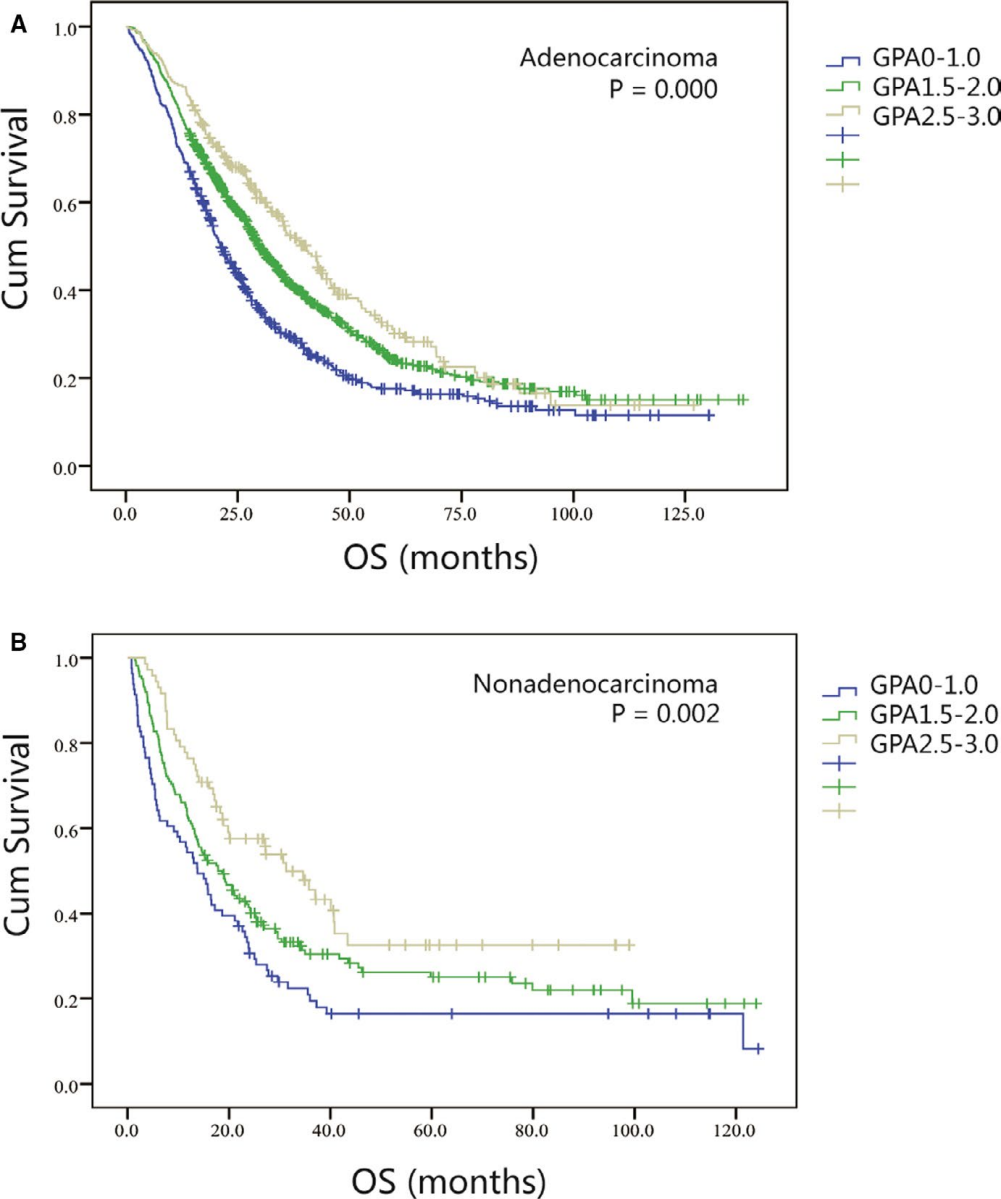
Kaplan‐Meier curves showing survival based on the adjusted DS‐GPA model for patients of NSCLC with BM. (A) Adenocarcinoma patients in the adjusted DS‐GPA model with scores of 0‐1.0, 1.5‐2.0, and 2.5‐3.0 had median OS of 21.2, 29.9, and 40.3 months (P=0.000). Adjacent classes stratification analysis had statistical significance (P=0.000 for 0‐1.0 vs 1.5‐2.0; and P=0.020 for 1.5‐2.0 vs 2.5‐3.0). (B) Non‐adenocarcinoma patients in the adjusted DS‐GPA model with scores of 0‐1.0, 1.5‐2.0, and 2.5‐3.0 had median OS of 13.8, 17.8, and 31.2 mont (P=0.002). Adjacent classes stratification analysis had different statistical P value (P=0.056 for 0‐1.0 vs 1.5‐2.0; and P=0.025 for 1.5‐2.0 vs 2.5‐3.0).

Figure 1A depicts the Kaplan‐Meier survival curves prepared using the adjusted DS‐GPA model for lung adenocarcinoma; these curves highlighted the overall difference between the groups (*p* = 0.000). Cases with scores of 0‐1.0, 1.5‐2.0, and 2.5‐3.0 had median OS of 21.2, 29.9, and 40.3 months, severally. Adjacent classes stratification analysis had statistical difference (*p* = 0.000 for 0‐1.0 vs 1.5‐2.0 and *p* = 0.020 for 1.5‐2.0 vs 2.5‐3.0). Its C‐index was 0.571. Figure 1B demonstrates the Kaplan‐Meier survival curves for lung non‐adenocarcinoma. They revealed the difference between the groups as a whole (*p* = 0.002). The corresponding median OS values were 13.8, 17.8, and 31.2 months. Adjacent classes stratification analysis showed different statistical *P* value (*p* = 0.056 for 0‐1.0 vs 1.5‐2.0 and *p* = 0.025 for 1.5‐2.0 vs 2.5‐3.0). Its C‐index was 0.599 (see Table 4 for details).

**Table 4:** Survival outcomes stratified by adjusted DS‐GPA and adjusted Lung‐molGPA for NSCLC with BM at diagnosis

Group	DS‐GPA		Lung‐molGPA	
	Pt.NO.(%)	MS(m)	Pt.NO.(%)	MS(m)
Adeno 0‐1.0	508 (32.0)	21.2	295 (18.6)	18.4
Adeno 1.5‐2.0	818 (51.5)	29.9	640 (40.3)	23.9
Adeno 2.5‐3.0	262 (16.5)	40.3	550 (34.6)	35.5
Adeno 3.5‐4.0	NA		103 (6.5)	49.0
Adeno overall	1588 (100)	28.0		
Non‐adeno 0‐1.0	81 (25.7)	13.8	76 (24.1)	10.3
Non‐adeno 1.5‐2.0	162 (51.4)	17.8	139 (44.1)	19.5
Non‐adeno 2.5‐3.0	72 (22.9)	31.2	91 (28.9)	27.1
Non‐adeno 3.5‐4.0	NA		9 (2.9)	37.0
Non‐adeno overall	315 (100)	18.7		

Abbreviations: Adeno, adenocarcinoma; BM, brain metastases; DS‐diagnosis specific; GPA, graded prognostic assessment; Lung‐mol, NSCLC with BM using molecular Markers; m, months; MS:median survival; NA, not applicable; NO, number; Non‐adeno, non‐adenocarcinoma; NSCLC, non‐small‐celllung cancer; Pt, patients.

**Figure 2:**
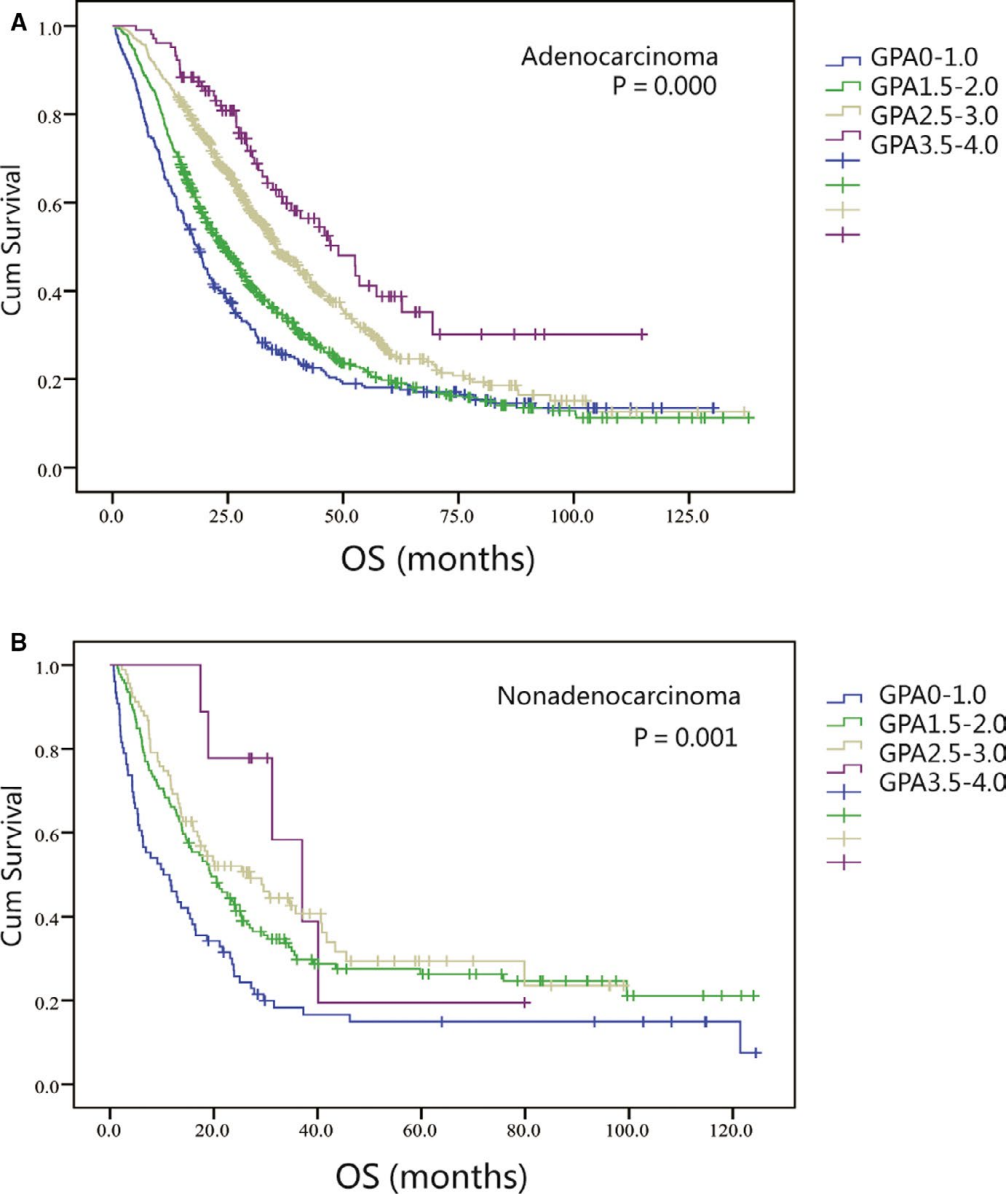
Kaplan‐Meier curves showing survival based on the adjusted Lung‐molGPA model for patients of NSCLC with BM. (A) Adenocarcinoma patients in the adjusted Lung‐molGPA model with scores of 0‐1.0, 1.5‐2.0, 2.5‐3.0, and 3.5‐4.0 had median OS of 18.4, 23.9, 35.5, and 49.0 months (P=0.000). Each adjacent classes stratification analysis had statistical significance (P=0.012 for 0‐1.0 vs 1.5‐2.0; P=0.000 for 1.5‐2.0 vs 2.5‐3.0 and P=0.007 for 2.5‐3.0 vs 3.5‐4.0). (B) Non‐adenocarcinoma patients in the adjusted Lung‐molGPA model with scores of 0‐1.0, 1.5‐2.0, 2.5‐3.0, and 3.5‐4.0 had median OS of 10.3, 19.5, 27.1, and 37.0 months (P=0.001). Only one adjacent class stratification analysis had statistical significance (P=0.003 for 0‐1.0 vs 1.5‐2.0; P=0.286 for 1.5‐2.0 vs 2.5‐3.0 and P=0.410 for 2.5‐3.0 vs 3.5‐4.0)

Figure 2A depicts the Kaplan‐Meier survival curves prepared using the adjusted Lung‐molGPA model for lung adenocarcinoma, which showed significant differences between groups on the whole (*p* = 0.000). Individuals with scores of 0‐1.0, 1.5‐2.0, 2.5‐3.0, and 3.5‐4.0 had median OS values of 18.4, 23.9, 35.5, and 49.0 months, severally. Each adjacent classes stratification analysis had statistical significance (*p* = 0.012 for 0‐1.0 vs 1.5‐2.0, *p* = 0.000 for 1.5‐2.0 vs 2.5‐3.0 and *p* = 0.007 for 2.5‐3.0 vs 3.5‐4.0). Its C‐index was 0.615. Figure 2B demonstrates it for lung non‐adenocarcinoma, and similarly indicates the difference between groups on the whole (*p* = 0.001). Individuals with scores of 0‐1.0, 1.5‐2.0, 2.5‐3.0, and 3.5‐4.0 had median OS values of 10.3, 19.5, 27.1, and 37.0 months, severally. However, stratification analysis showed statistical difference in only one class (*p* = 0.003 for 0‐1.0 vs 1.5‐2.0, *p* = 0.286 for 1.5‐2.0 vs 2.5‐3.0; and *p* = 0.410 for 2.5‐3.0 vs 3.5‐4.0). Its C‐index was 0.621 (see Table 4 for detailed).

## 4 DISCUSSION

In this retrospective study, we created a data set of 1903 NSCLC patients with BM from the southwestern Chinese population; its median OS was 27.0 months, and score of 3.5‐4.0 reached a median OS of 49.0 months in the adjusted Lung‐molGPA model for lung adenocarcinoma. The adenocarcinoma had better prognosis than the non‐adenocarcinoma (median OS: 28.0 months vs 18.7 months, *p* < 0.001). The adjusted Lung‐molGPA model was more accurate in predicting survival of the adenocarcinoma than the adjusted DS‐GPA model (C‐index: 0.615 vs 0.571), but it failed to estimate the prognosis for the non‐adenocarcinoma (*p* = 0.286, 1.5‐2.0 vs 2.5‐3.0; *p* = 0.410, 2.5‐3.0 vs 3.5‐4.0).

The median OS obtained in the current study was not only longer than the 12.0 months and 5.4 months reported in the study by Sperduto^13^ and Nieder,^14^ but was also longer than the 11.3 months and 14.0 months of two Chinese cohorts.^15^, ^16^ Similar to the study by Li and Fan,^15^, ^16^ the start point of OS in our study was calculated from BM diagnosis, but two other studies started calculating OS from BM treatment.^13^, ^14^ In the current cohort, there were 23.8% individuals still surviving to the last follow‐up, 57% cases with KPS ≥90, 42.8% patients at EGFR or ALK positive, and the vast majority of patients were lung adenocarcinoma. This discrepancy may explain why patients in our study had longer survival than those in other studies. According to a previous report on NSCLC, metastasis organ numbers at initial diagnosis was an independent prognosis factor.^18^ There had 26.8% patients without ECM, and 51.8% patients had only 1‐2 ECM organ numbers and this may be another explanation for the results we obtained. In studies conducted in North America^13^ and Europe,^14^ adenocarcinoma patients with BM and Lung‐molGPA model score of 3.5‐4.0 had a median survival time of 46.8 months and 25.0 months. The corresponding OS value in our study was 49.0 months, it was longer than 17.0 months seen in the study by Li,^15^ but less than of 62.0 months seen in the study by Fan.^16^ In Fan's^16^ Lung‐molGPA model, the gene mutation state was used as the stratification analysis factor, but the pathological subtype as it in the Sperduto's^13^ and our study. The study by Li only estimated survival of the adenocarcinoma with BM, and included patients who received the best supportive care, but them were excluded in our study.^15^ In the European study, including those patients with intermediate or poor prognosis could not undergo aggressive local therapies.^14^ This variation of stratification and selected cases may explain why our survival result was similar to that of Sperduto's^13^ study, but shorter than the survival seen in the study by Fan^16^ and longer than that seen in the studies by Li^15^ and Nieder.^14^

In univariate analysis, several factors were found to be associated with survival, including sex, smoking status, KPS, BMI, N‐staging, ECM, ECM organ numbers, BM numbers, histology type, and gene mutation status. Moreover, those factors were correlated with patients prognosis such as KPS, BMI, smoking status, N‐staging, ECM organ numbers, BM numbers, histology type, and gene mutation status in multivariate analysis. Interestingly, as a known predictor of survival, even when the cutoff value was adjusted to the median age of 57 years in this cohort, age was found to be correlated with the prognosis neither of the univariate and multivariate analysis, which was not consistent with the results obtained in previous studies.^8^, ^9^, ^13^, ^14^, ^15^, ^16^ There is perhaps an inherent selection bias in this retrospective study. Another reason for this may be that patients who are EGFR or ALK positive, which was over 40% of the cohort, received target therapy, which might have affected the results of the Log‐rank and Cox regression analysis and decreased the weight of age. Inconsistent with the results of Li's^15^ and Fan's^16^ studies, we found a correlation between BM numbers and prognosis in the Chinese cohort, similar to what was found in studies in Northern America and Europe.^13^, ^14^ In a European study, underweight patients having lung cancer with BM at diagnosis had a shorter median OS than normal and overweight patients.^19^ The corresponding OS values were 20.8, 26.5, and 29.3 months for patients with NSCLC and BM in our study, and these were longer than those of the European cohort.^19^ This is partly attributable to there being SCLC cases in their study. It revealed that BMI ≥24.0 was a good prognostic factor for patients with NSCLC‐BM. Based on the presence or absence of ECM, we found that the ECM organ numbers had a significant relationship with survival. Patients with 1‐2 ECM organ numbers had longer survival times than those with >3 (26.9 months vs 18.8 months, *p* < 0.001). Perhaps, in addition to ECM, determining the ECM organ numbers is a more important predictor of survival for patients with NSCLC and BM. Thus, more research is needed to further evaluate the role of BM numbers, especially BMI and ECM organ numbers, in the prognoses of NSCLC with BM to guide prospective clinical trial stratification and prognostic assessments.

As a valuable independent predictor for the prognosis of NSCLC with BM, driver gene alteration has been incorporated into the prognosis assessment models that are widely used.^8^, ^13^, ^14^, ^15^, ^16^, ^20^ In the adjusted Lung‐molGPA model for adenocarcinoma, patients with Lung‐molGPA scores of 0‐1.0, 1.5‐2.0, 2.5‐3.0, and 3.5‐4.0 had median OS of 18.4, 23.9, 35.5, and 49.0 months, which were longer than those obtained in the studies by Li,^15^ Sperduto,^13^ and Nieder.^14^ The adjusted Lung‐molGPA model estimates of the prognosis of adenocarcinoma with BM, both via whole (*p* = 0.000, C‐index = 0.615) and adjacent stratification analyses (*p* = 0.012 for 0‐1.0 vs 1.5‐2.0, *p* = 0.000 for 1.5‐2.0 vs 2.5‐3.0 and *p* = 0.007 for 2.5‐3.0 vs 3.5‐4.0) were statistical significance. The adjusted DS‐GPA model (*p* = 0.000, C‐index = 0.571) can also predict the prognosis of lung adenocarcinoma with BM, but the adjusted Lung‐molGPA model (*p* = 0.000, C‐index=0.615) was a better estimating tool. Consideration that 16.8% of lung non‐adenocarcinoma individuals with BM were EGFR or ALK positive, we used the adjusted Lung‐molGPA model to assess their survival. Although there was a statistical significance among groups as a whole (*p* = 0.001), two adjacent classes was not found to be statistically significant in the stratified analysis (*p* = 0.286 for 1.5‐2.0 vs 2.5‐3.0 and *p* = 0.410 for 2.5‐3.0 vs 3.5‐4.0). This maybe correlated with the uneven stratification of cases and the fact that only nine patients had score of 3.5‐4.0. It manifested once again that gene mutation status was not a suitable prognostic factor for lung non‐adenocarcinoma with BM. Interestingly, even in the adjusted DS‐GPA model, there was statistical difference in the whole comparison (*p* = 0.002), but one adjacent classes analysis still had close to no statistical significance (*p* = 0.056 for 0‐1.0 vs 1.5‐2.0). Overall, the adjusted DS‐GPA model was still a more suitable model for predicting the prognosis of lung non‐adenocarcinoma with BM (*p* = 0.002, C‐index =0.599).

In the current study, there are some shortcomings. First, it is a retrospective study, with inherent selection bias and the treatment factor on prognosis was not considered. Second, it excluded those patients who only received best supportive care, because the number of cases was so small. Third, identification of gene mutations principally by detecting tumor lesions in the plasma or elsewhere outside the brain does not reflect the mutation status in the intracranial metastases due to the existence of the blood‐brain barrier (BBB) and tumor heterogeneity.^21^, ^22^ In particular, tumor heterogeneity in gene mutation in time and space is very important for the prognosis of lung cancer patients.^23^, ^24^, ^25^

## 5 CONCLUSIONS

The current study proved the applicability of the adjusted Lung‐molGPA model in a Chinese cohort of adenocarcinoma NSCLC with BM. Simultaneously, the adjusted DS‐GPA model was still a good user‐friendly tool to predict survival for non‐adenocarcinoma patients. Thus, more studies are needed to estimate the prognosis for patients of NSCLC with BM to improve future clinical trial stratification and help doctors make optimal treatment decisions in clinical practice.

## CONFLICT OF INTEREST

All authors have read and approved the final manuscript. No authors reported a conflict of interest related to the study.

## AUTHORS’ CONTRIBUTIONS

TYZ: Project conceptualization, cases collection, data curation, methodology, original draft writing, and funding acquisition. YZ: Project conceptualization, cases collection, data analysis, drawing processing, methodology, and draft review and editing. LZ: Project conceptualization, cases collection, data analysis, methodology, original draft writing, and draft review and editing. SSD: Cases collection and drawing processing. MJH: Cases collection and methodology. YCL: Cases collection and drawing processing. YML: Project conceptualization and cases collection. YL: Project design, data curation, formal analysis, funding acquisition, methodology, supervision, and writing‐review and editing. Other authors: cases collection. All authors read and approved the final manuscript.
